# Dapsone induced hemolysis in a patient with Hansen’s disease and G6PD deficiency: A preventable peril

**DOI:** 10.1002/ccr3.5700

**Published:** 2022-04-26

**Authors:** Arnab Kumar Ghorui, Pratap Kumar Patra

**Affiliations:** ^1^ Department of Pediatrics All India Institute of Medical Sciences Patna India

**Keywords:** dapsone, G6PD deficiency, Hansen's disease

## Abstract

Although under‐reported, hemolytic anemia is common with dapsone‐containing regimen in leprosy. It is prudent to screen for underlying G6PD deficiency in boys before administering dapsone to prevent potentially life‐threatening episode of intravascular hemolysis in children with leprosy.

## Abstract 2

Hansen's disease is a significant public health issue in many countries. Underlying G6PD deficiency should be ruled out before prescribing dapsone containing regimen in boys to prevent catastrophic intravascular hemolysis.

An 11‐year‐old boy was noted to have a hypopigmented patch over the left cheek with diminished sensation (Figure 1A). Thickening of bilateral ulnar nerves was additional finding. Hansen's disease was diagnosed, and he was treated with clofazimine, dapsone, and rifampicin at a local healthcare facility. After 3 days, he developed fever, yellow discoloration of eyes, and cola‐colored urine (Figure 1B). On examination, severe pallor, icterus, and splenomegaly were noted. Investigation revealed hemoglobin 58 g/L, indirect bilirubin 5.3 mg/dL, serum lactate dehydrogenase 5619 U/L (Normal <170 U/L), and aspartate aminotransferase 210.9 U/L (Normal <40 U/L). The level of G6PD was significantly reduced. He required two units packed red blood cell transfusion along with 1.5 times maintenance fluid and injection furosemide. Further, dapsone was replaced with ofloxacin. He recovered after 7 days of hospitalization.

**FIGURE 1:**
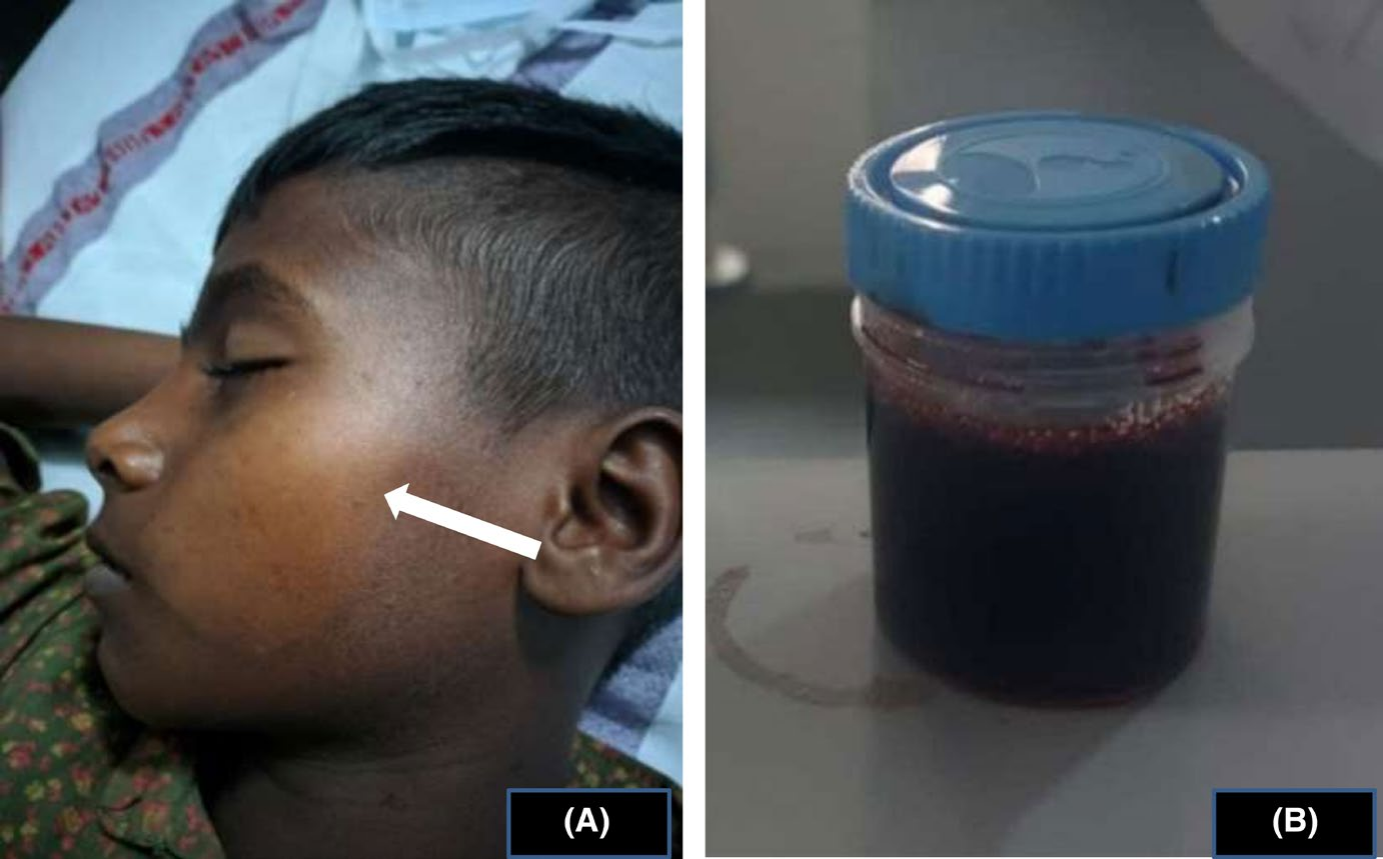
(A) Hypopigmented lesion over the left cheek. (B) Image of cola‐colored urine

Although under‐reported, hemolytic anemia is common during the treatment of leprosy and related to N‐hydroxy metabolite of dapsone.^1^ However, concomitant G6PD deficiency enhances the risk of intravascular hemolysis, which may be severe, and a cause of significant morbidity. Furthermore, as the prevalence of G6PD deficiency in India varies from 2.3% to 27%, there may be a chance that both conditions exist simultaneously.^2^ Therefore, even in a resource constraint setting with a significant prevalence of G6PD deficiency, it is prudent to screen for underlying G6PD deficiency in boys before oxidant medications are to be administered.

## CONFLICTS OF INTEREST

None.

## AUTHOR CONTRIBUTION

Arnab Ghorui contributed to patient management, data collection, and manuscript drafting. Pratap Kumar Patra contributed to manuscript drafting, editing, and final approval.

## ETHICAL APPROVAL

None.

## CONSENT

Written consent has been obtained from the parents to publish this image.

## Data Availability

Data are available and will be shared on request.
